# Mixed Functionalization of Organic Ligands in UiO-66: A Tool to Design Metal–Organic Frameworks for Tailored Microextraction

**DOI:** 10.3390/molecules24203656

**Published:** 2019-10-10

**Authors:** Gabriel González-Rodríguez, Iván Taima-Mancera, Ana B. Lago, Juan H. Ayala, Jorge Pasán, Verónica Pino

**Affiliations:** 1 Departamento de Química, Unidad Departamental de Química Analítica, Universidad de La Laguna (ULL), Tenerife, 38206 La Laguna, Spain; alu0100995312@ull.edu.es (G.G.-R.); ivan.taima.13@ull.edu.es (I.T.-M.); jayala@ull.edu.es (J.H.A.); 2 Laboratorio de Rayos X y Materiales Moleculares (MATMOL), Departamento de Física, Universidad de La Laguna (ULL), Tenerife, 38206 La Laguna, Spain; alagobla@ull.edu.es; 3 University Institute of Tropical Diseases and Public Health, Universidad de La Laguna (ULL), Tenerife, 38206 La Laguna, Spain

**Keywords:** metal–organic frameworks, dispersive miniaturized solid-phase extraction, mixed functionalization, interactions MOF–analyte, UiO-66

## Abstract

The mixed-ligand strategy was selected as an approach to tailor a metal–organic framework (MOF) with microextraction purposes. The strategy led to the synthesis of up to twelve UiO-66-based MOFs with different amounts of functionalized terephthalate ligands (H-bdc), including nitro (-NO_2_) and amino (-NH_2_) groups (NO_2_-bdc and NH_2_-bdc, respectively). Increases of 25% in ligands were used in each case, and different pore environments were thus obtained in the resulting crystals. Characterization of MOFs includes powder X-ray diffraction, infrared spectroscopy, and elemental analysis. The obtained MOFs with different degrees and natures of functionalization were tested as sorbents in a dispersive miniaturized solid-phase extraction (D-µSPE) method in combination with high-performance liquid chromatography (HPLC) and diode array detection (DAD), to evaluate the influence of mixed functionalization of the MOF on the analytical performance of the entire microextraction method. Eight organic pollutants of different natures were studied, using a concentration level of 5 µg· L^−1^ to mimic contaminated waters. Target pollutants included carbamazepine, 4-cumylphenol, benzophenone-3, 4-tert-octylphenol, 4-octylphenol, chrysene, indeno(1,2,3-cd)pyrene, and triclosan, as representatives of drugs, phenols, polycyclic aromatic hydrocarbons, and disinfectants. Structurally, they differ in size and some of them present polar groups able to form H-bond interactions, either as donors (-NH_2_) or acceptors (-NO_2_), permitting us to evaluate possible interactions between MOF pore functionalities and analytes’ groups. As a result, extraction efficiencies can reach values of up to 60%, despite employing a microextraction approach, with four main trends of behavior being observed, depending on the analyte and the MOF.

## 1. Introduction

Metal–organic frameworks (MOFs) are having enormous success as novel sorbent materials in analytical solid-phase extraction (SPE) approaches, particularly when performing in dispersive and miniaturized modes (D-µSPE) [1,2,3,4,5]. The synergies of MOFs’ features, such as their impressive surface area, synthetic tuneability, and chemical stability [6,7], and those of D-µSPE, such as method simplicity and a high efficiency [8,9], are among the reasons justifying the high number of recent studies in the field.

A step forward in ensuring the true expansion of MOFs as competitive materials for D-µSPE requires not only the assurance of a better performance than that resulting from commercial materials (exhaustive comparison and inter-laboratory validation) [5,10], but also deep evaluation of the main factors of MOFs justifying the improved analytical performance for target compounds [11,12]. Gaining an understanding of the process can serve as the basis of proper MOF design.

A number of studies using MOFs in D-µSPE have pointed out the complexity of the systems, indicating the pore environment, pore size, and pore aperture widths of the MOF as the most influential factors, together with a clear influence of the metal nature (particularly the presence of unsaturated metal sites) [11]. Lirio et al. also pointed out the influence of the metal, particularly the radius of the metal [13]. Taima-Mancera et al. showed the positive effects of incorporating polar functionalities in the organic ligand of the MOFs used in D-µSPE when intending to extract polar analytes of a small size [12], with this idea having been further expanded by Boontongto et al. to other application studies for polar analytes [14].

Computational and modeling studies are also powerful tools for evaluating the adequacy of MOFs for different applications, but have hardly been tested for MOFs in D-µSPE. Therefore, the majority of studies have been devoted to the evaluation of gas storage applications of MOFs or in catalysis studies [15,16,17]. However, it is fair to mention the studies of Gao et al. [18], which computationally selected the MOF MIL-53(Al) as adequate material for a D-µSPE method to determine a group of estrogens and glucocorticoids, and proved it with experimental studies.

In spite of the abovementioned studies that have tried to provide insights linked to the nature of the MOFs to improve their performance for target analytes in D-µSPE, most MOFs in reported applications are archetypical MOFs. This is particularly true for those that are currently commercialized, such as MIL-53(Al) [11] HKUST-1 [19], and MIL-100(Fe) [20].

Currently, Zr-based MOFs are widely studied in a number of fields (not exclusively in D-µSPE) because of their high chemical stability [21,22]. Among Zr-based MOFs, UiO-66(Zr) and UiO-66-type MOFs have been studied the most, given their superior chemical and hydrothermal stability, together with their simple (and mild) preparation [23] and green aspects [24]. Therefore, they have appeared as sorbents in a number of recent D-µSPE studies [12,25,26,27].

Regarding the modifications of UiO-66 to obtain a number of derivatives, it is interesting to mention the mixed-linker approach, which consists of incorporating two or more linkers with similar sizes, but different functional groups [28]. In this way, the resulting framework will present the properties modulated by the relative amounts of functional groups incorporated [29]. Cohen et al. were the first to incorporate -Br and -NH_2_ functionalities into the organic ligand (1,4-benzenedicarboxylic acid) of UiO-66 [30], and obtained a more thermally-stable derivative than the neat UiO-66. The incorporation of different contents of -NH_2_ functionalization into UiO-66 has also been proposed, not only resulting in a thermally-stable superior material, but also permitting the porosity to be tuned by varying the ratios of non-functionalized ligand *versus* -NH_2_-functionalized ligand [31].

Given these considerations, the current study intends to prepare and characterize UiO-66 derivatives incorporating different contents of non-functionalized and functionalized-organic ligand—including -NH_2_ and -NO_2_ groups—in the MOF structure through the mixed-linker approach. As a second goal, it pursues an evaluation of the influence of such modifications in the resulting material when used as a sorbent in a D-µSPE method for different target analytes in water. The selected analytes present a low to high size (to evaluate their influence when entering or when not entering the MOFs’ pores), while incorporating or not incorporating polar groups in their structures (to evaluate possible interactions between MOF pore functionalities and analytes’ groups).

## 2. Experiment

### 2.1. Chemicals, Reagents, and Materials

Six out of the eight target analytes were obtained as solid products from Sigma-Aldrich (Steinheim, Germany): carbamazepine (Cbz, 99.0%), 4-cumylphenol (CuP, 99%), 4-tert-octylphenol (t-OP, 97%), 4-octylphenol (OP, 99%), benzophenone-3 (BP-3, 99.5%), and chrysene (Chy, 98%). The remaining two target analytes, indeno(1,2,3-cd)pyrene (Ind) and triclosan (Tr), were purchased separately as standard solutions, with a concentration of 10 mg·L^−1^ in acetonitrile (ACN), by Dr. Ehrenstorfer GmbH (Augsburg, Germany). Chemical structures of the studied analytes are included in Table 1. A standard solution containing all eight target compounds was prepared in ACN Chromasolv^TM^ liquid chromatography (LC) grade, purchased from Honeywell Fluka^TM^ (Seelze, Germany), at a concentration of 5 mg·L^−1^, and stored at 4 °C. Aqueous working standard solutions at 5 µg·L^−1^ containing all compounds were utilized in the D-µSPE method.

Reagents included in the synthesis of the UiO-66 MOF and its functionalized derivatives were ZrCl_4_ (98%), HCl (37%, *v*/*v*), 1,4-benzenedicarboxylic acid (H-bdc, 98%), 2-amino-1,4-benzenedicarboxylic acid (NH_2_-bdc, 99%), and 2-nitro-1,4-dicarboxylic acid (NO_2_-bdc, ≥99%), purchased from Sigma-Aldrich. Dimethylformamide (DMF, ≥99.5%) was acquired from Merck KGaA (Darmstadt, Germany), and methanol (≥99.8%) was purchased from PanReac AppliChem (Barcelona, Spain).

The synthesis of MOFs required Teflon (PTFE^®^) solvothermal reactors of a 45 mL capacity and stainless-steel autoclaves, all from Parr Instrument Company (Moline, IL, USA).

Ultrapure water (Milli-Q, ultrapure grade) was obtained through the purification system A10 MilliPore (Watford, UK). High-performance liquid chromatography (HPLC) mobile phases were prepared with ultrapure water and ACN Chromasolv^TM^ LC-MS grade. Both phases were filtered with 0.45 μm Durapore^®^ membrane filters of Sigma-Aldrich.

Additionally, 0.2 μm polyvinylidene fluoride (PVDF) syringe filters Whatman^TM^, purchased from GE Healthcare (Buckinghamshire, UK), were used when filtrating desorption solvents after application of the D-µSPE method. The microextraction method also required glass centrifuge tubes of a 28 mL capacity from Pyrex^®^ (Corning Inc., Staffordshire, UK), with a size of 10 × 2.6 cm.

### 2.2. Instrumentation

In the D-µSPE procedure, a vortexer from Reax-Control Heidolph^TM^ GmbH (Schwabach, Germany) and the centrifuge model 5720 Eppendorf^TM^ (Hamburg, Germany) were utilized.

The HPLC model 1260 Infinity was purchased from Agilent Technologies (Santa Clara, CA, USA). A Rheodyne injection valve with an injection loop of 20 µL, supplied by Supelco (Bellefonte, PA, USA), was included in the system. The chromatographic separation used an ACE Ultra Core 5 SuperC18 (5 µm, 150 × 4.6 mm) analytical column, obtained from Symta (Madrid, Spain), with the safeguard column Pelliguard LC-18 purchased from Supelco. Ultrapure water and ACN were employed as mobile phases using a linear gradient at a constant flow rate of 1 mL·min^−1^. The chromatographic method started at 50% (*v*/*v*) of ACN for 5 min and then increased up to 80% (*v*/*v*) for 2 min and up to 83% (*v*/*v*) in the next 2.5 min, before finally reaching 100% (*v*/*v*) of ACN in the next 3.5 min.

The detection of analytes was achieved with a diode array detection (DAD) 1260 Infinity model, purchased from Agilent Technologies. The quantification wavelengths of the DAD were set at 254 nm for Ind; 270 nm for Chy; 280 nm for CuP, t-OP, and OP; and 289 nm for Cbz, BP-3, and Tr.

The Universal UF30 oven, supplied by Memmert (Schwabach, Germany), was used in MOF synthesis.

All the MOFs were characterized by powder X-ray diffraction using a PANalytical Empyrean diffractometer (Eindhoven, The Netherlands) with Cu Kα radiation (λ = 1.5418 Å) and operating with Bragg–Brentano geometry. Measurements were carried out at room temperature in the range from 5.01° to 80.00° (0.02° steps), with a total exposure time of 12 min.

A Gemini V2365 model, supplied by Micromeritics (Norcross, GA, US), was used to measure the nitrogen adsorption isotherms with a surface area analyzer at 77 K in the range 0.02 ≤ P/P_0_ ≤ 1.00. The Brunauer, Emmett and Teller (BET) method was used to calculate the surface area.

An infrared spectroscopy instrument with Fourier transformed (FT-IR) model IFS 66/S from Bruker (MA, US) was used.

Elemental analyses (C, H, N) were carried out with the elemental analyzer CNHS Flash EA 1112 from Thermo Fisher Scientific (Massachusetts, MA, USA).

### 2.3. Procedures

#### 2.3.1. Synthesis of MOFs

**Scheme 1:**
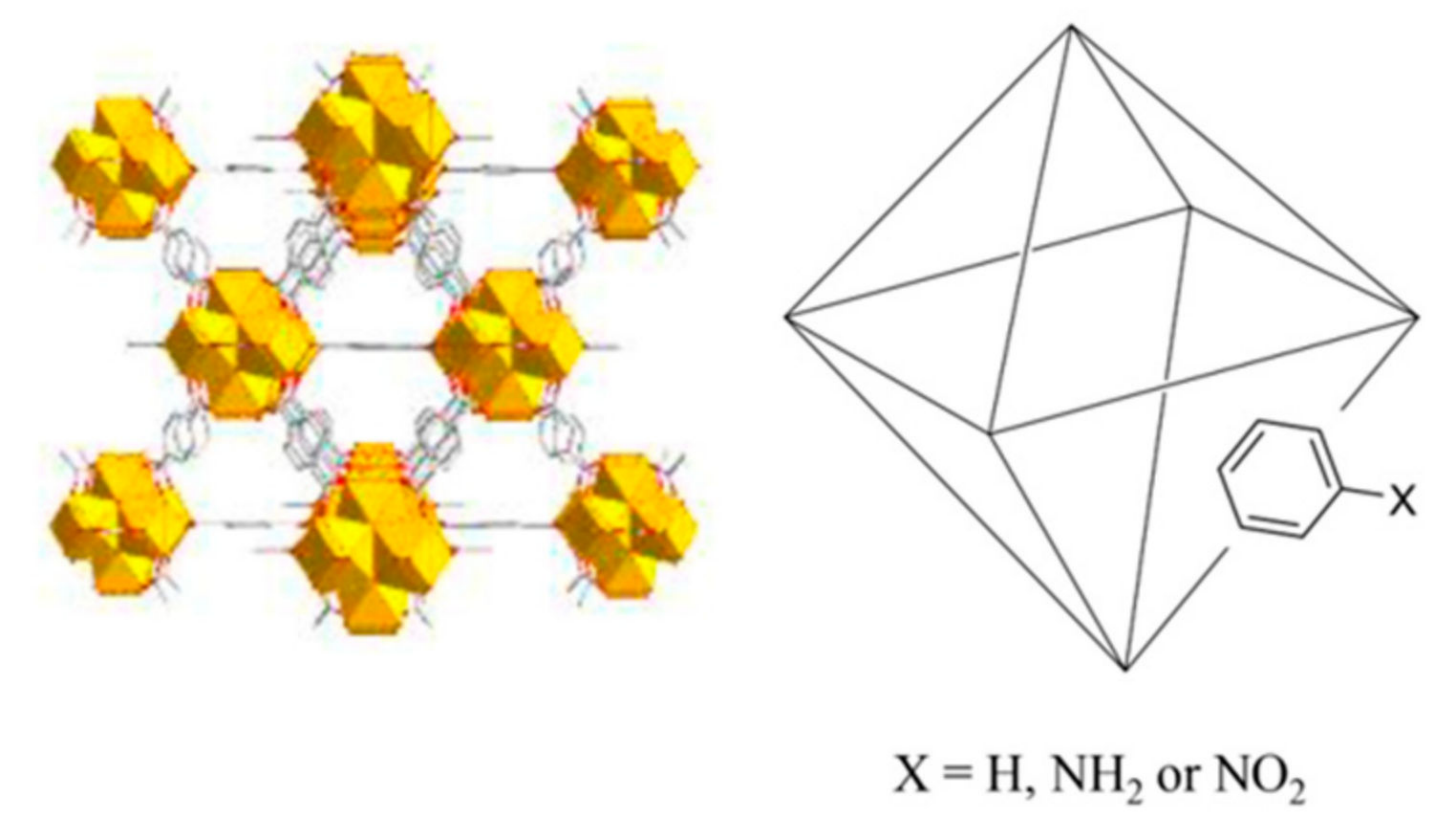
Schematic structure of UiO-66 and its derivatives.

The synthesis of UiO-66 and its functionalized variants (Scheme 1) followed the procedure reported by Taima-Mancera et al. [12,32]. Briefly, UiO-66 required 233 mg of ZrCl_4_ (1 mmol) and 246 mg of H-bdc (1.5 mmol). These reagents were dissolved in 15 mL of DMF, with 1 mL of concentrated HCl as a synthetic modulator. The resulting solution was heated in a solvothermal reactor at 150 °C for 24 h. Once cooled at room temperature, the obtained solid was filtered, washed twice with DMF (24 h each), filtered again, and then washed with methanol (24 h). Finally, the product was heated at 150 °C for one day in order to activate the MOF.

**Figure 1:**
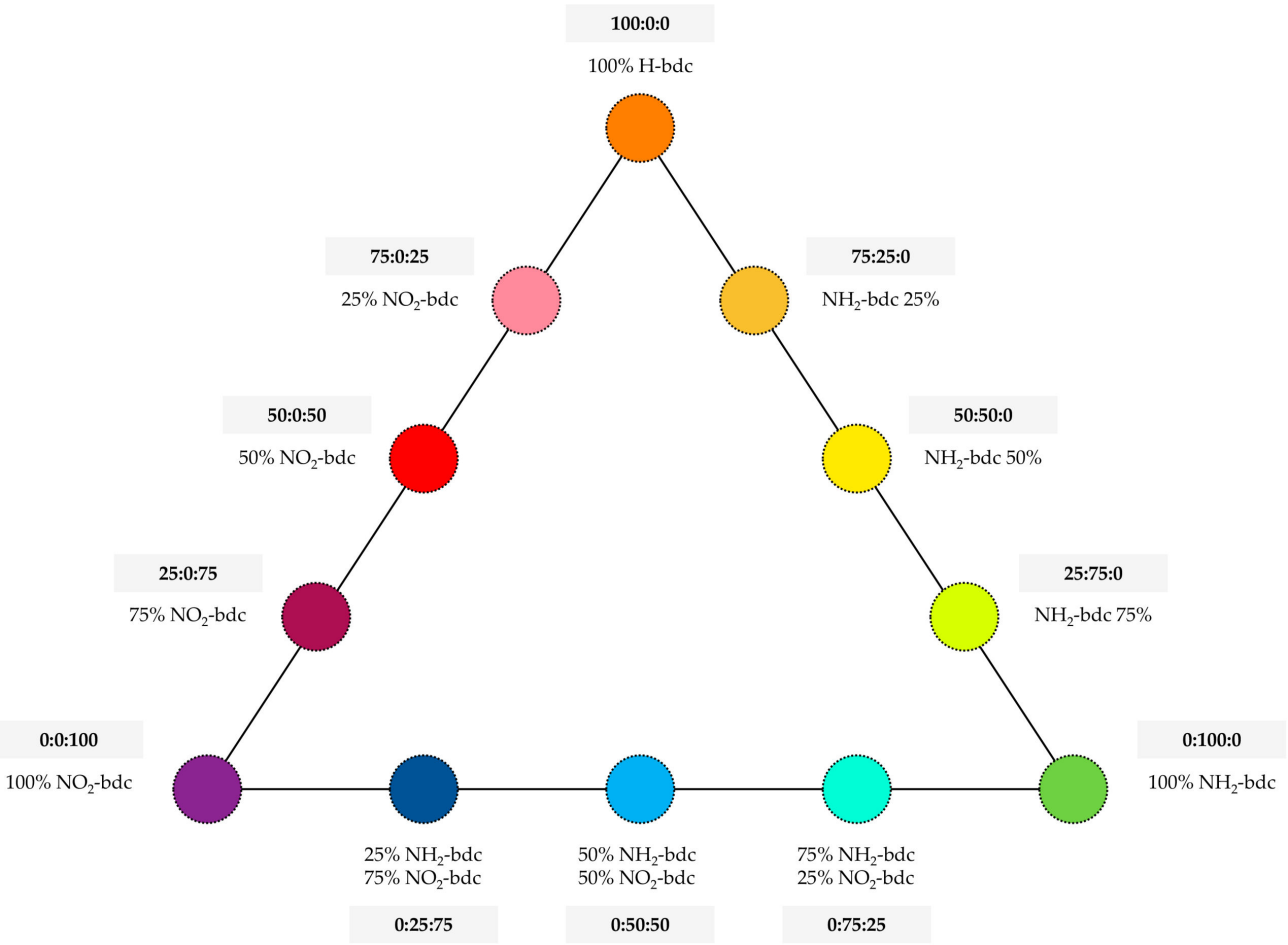
UiO-66-based metal–organic frameworks (MOFs) prepared with different contents of functional groups, labeled in the triangular diagram as percentages of functionalized terephthalate ligands.

UiO-66-functionalized variants were prepared analogously, replacing H-bdc with the equivalent molar amounts of NH_2_-bdc or NO_2_-bdc, depending on the specific MOF under preparation. In this sense, different ratios of NH_2_-bdc, NO_2_-bdc, and H-bdc were included in the synthetic approach, with the purpose of obtaining MOFs with different amounts of functionalities. The final set includes the preparation of up to 12 derivatives of the UiO-66 MOF, with the specific contents included in Figure 1.

#### 2.3.2. Dispersive Miniaturized Solid-Phase Extraction (D-µSPE) Method

The extraction procedure followed our previous studies on the use of the MOF UiO-66 for determining endocrine disrupting chemicals using D-µSPE-HPLC-DAD [12], but minimizing the initial content of MOF. Therefore, the current study required the use of a lower amount of MOF and employed 10 mg rather than 20 mg. In summary, 10 mg of the UiO-66-based MOF were used when analyzing 20 mL of an aqueous standard containing target analytes. The microextraction took place in Pyrex^®^ tubes subjected to 3 min of vortex, to increase the strength of the sorbent (MOF)–analyte interactions. Afterwards, phases were separated by centrifugation (2504× *g* for 5 min), followed by separation of the supernatant with a Pasteur pipette. Desorption took place using 500 μL of ACN under 5 min of vortex agitation, followed by 5 min of centrifugation (2504× *g*). Finally, the desorption solution was filtered through 0.2 μm PVDF syringe filters before HPLC injection. The entire procedure is schematized in Figure S1 of the ESM.

## 3. Results and Discussion

### 3.1. Characterization of the UiO-66-Based MOFs Obtained with the Mixed-Linker Approach

Complete characterization by powder X-ray diffraction, nitrogen adsorption isotherms, elemental analysis, and infrared spectroscopy took place for the synthesized and activated UiO-66-based MOFs (Figure 1). Powder X-ray diffraction patterns were obtained in order to verify the crystalline structure of all MOFs through a comparison with the simulated one for the UiO-66 [33]. Furthermore, N_2_ adsorption isotherms were used for the calculation of Brunauer–Emmett–Teller (BET) surface areas. Likewise, infrared spectra were utilized to identify nitro- and amino-functional groups in the MOFs, and the elemental analysis was carried out to evaluate whether the degree of functionalization was correctly introduced in the resulting MOFs.

All powder X-ray diffraction patterns are included in Figures S2–S4 of the ESM. It is important to highlight that all obtained UiO-66-based MOFs using the current approach were crystalline and topologically identical.

The BET surface areas were calculated from the nitrogen adsorption isotherms for all MOFs, and the obtained data is shown in Table S1 of the ESM. The trend shows a decrease in the surface area with the increased degree of functionalization. For example, UiO-66 showed a BET surface area value of 1175 m^2^ g^−1^, whereas the increasing content of the amino group as functionalization (from 25% to 100%) showed decreasing values, down to 678 m^2^ g^−1^. For the nitro group as UiO-66 functionalization, values also decreased from 717 m^2^ g^−1^ at 25% to 604 m^2^ g^−1^ at 100%. If considering the amino/nitro group mixed functionalization in the UiO-66-based MOFs, the values range from the 100% nitro group to 100% amino group.

**Figure 2:**
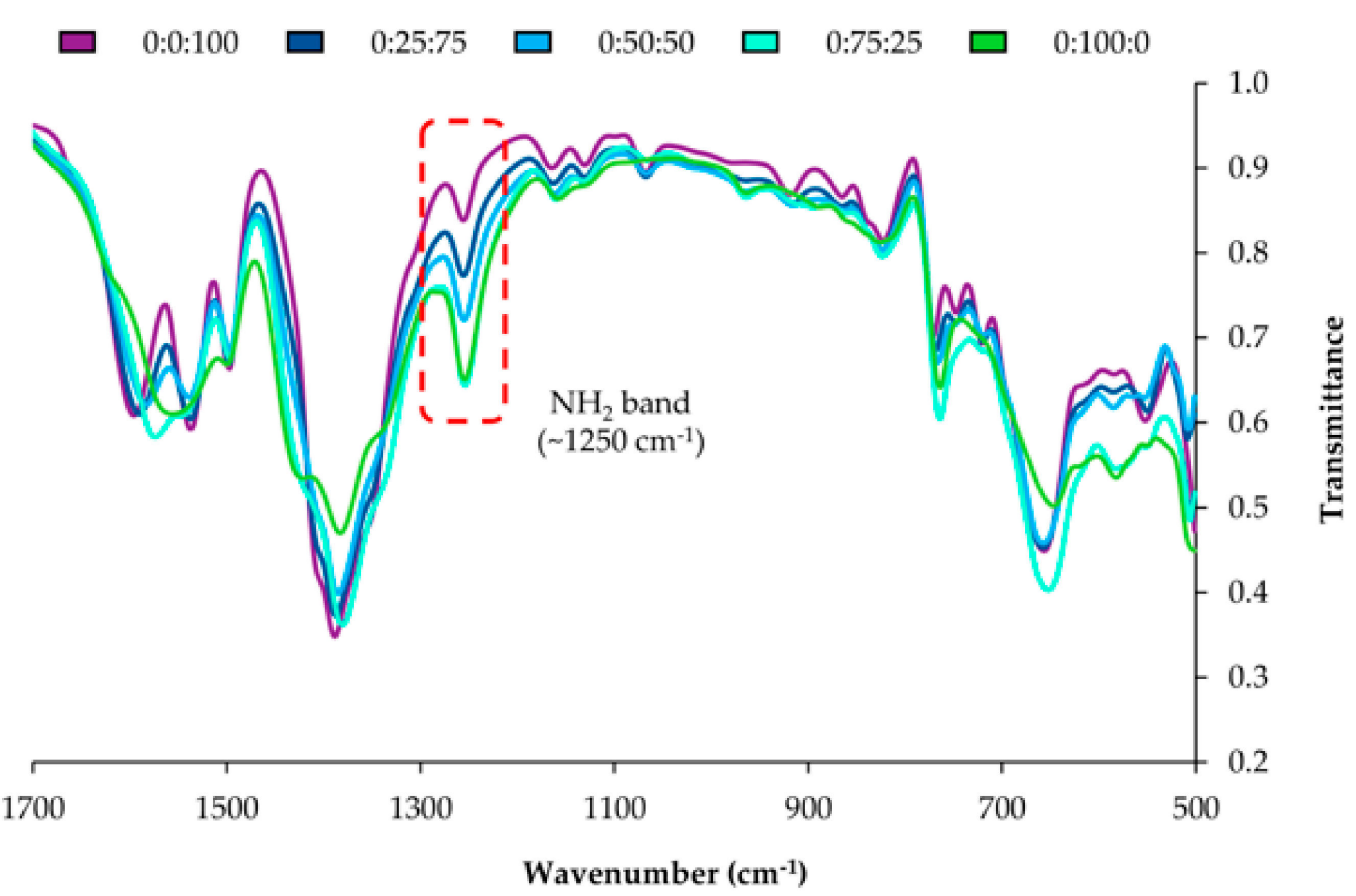
Zoom in the infrared spectra of the NO_2_-bdc/NH_2_-bdc series, from 500 to 1700 cm^−1^. The code H-bdc: NH_2_-bdc:NO_2_-bdc is included for each MOF.

Figure S5 of the ESM includes the infrared spectra for the NH_2_-bdc:NO_2_-bdc series, whereas Figure 2 shows a zoom from 500 to 1700 cm^−1^ for such series. The FT-IR spectra of the MOFs display features corresponding to the bdc ligand and to the amino or nitro groups present in each MOF. It can be clearly observed that the intensity of the IR band at 1257 cm^−1^ (attributed to the symmetric in-plane bending or deformation mode of the -NH_2_ group) increases with the content of NH_2_-bdc, thus supporting the proper inclusion of the amino functionality [34] and its increasing content in the series. Moreover, a broad band at 3367 cm^−1^ is observed for amino derivatives (N–H stretching modes). This band is more defined when the content of amino groups increases, as occurred for the 25:75:0 and 0:100:0 MOFs in Figure S6 of the ESM. Furthermore, the intensity of the band associated with the nitro functionalization at 1546 cm^−1^ (N–O stretching modes) [34] rises when increasing the amount of nitro groups in the MOF.

The formulae proposed for the different functionalized compounds are supported by the CHN elemental analysis. Table S2 of the ESM presents elemental analysis data, where the match between the experimental data and the calculated data can be observed. A higher degree of functionalization with both NH_2_-bdc and NO_2_-bdc implies an increase in the nitrogen content.

### 3.2. Analytical Performance of the D-µSPE-HPLC-DAD Method When Using Derivatives of UiO-66

All MOFs were used as sorbents in the D-µSPE-HPLC-DAD method following the conditions described in Section 2.3.2, with experiments carried out in triplicate. The target compounds included eight endocrine disrupting chemicals, specifically four small-sized analytes with polar functional groups in their structures (Cbz, CuP, Tr, and BP-3) and four heavier compounds (t-OP, OP, Chy, and Ind), two of which had no polar group in their structures. These common pollutants in water were selected in the current study to cover a wide range of possible MOF–analyte interactions. The main purpose was to evaluate the effects that mixed functionalization in the MOFs exerted on extraction efficiencies for these analytes in water. To extract proper conclusions, it is important to take into account the following considerations: (i) the amino group in the MOF can act as a hydrogen bond donor and it is an electron donating group towards the terephthalate ring, but the nitro group is a hydrogen bond acceptor and an electron withdrawal group; (ii) the pore window of the MOF reduces when the degree of functionalization increases; and (iii) the functionalization groups are mainly located in the pores, but they are also present in the external surface of the MOF crystallites.

**Figure 3:**
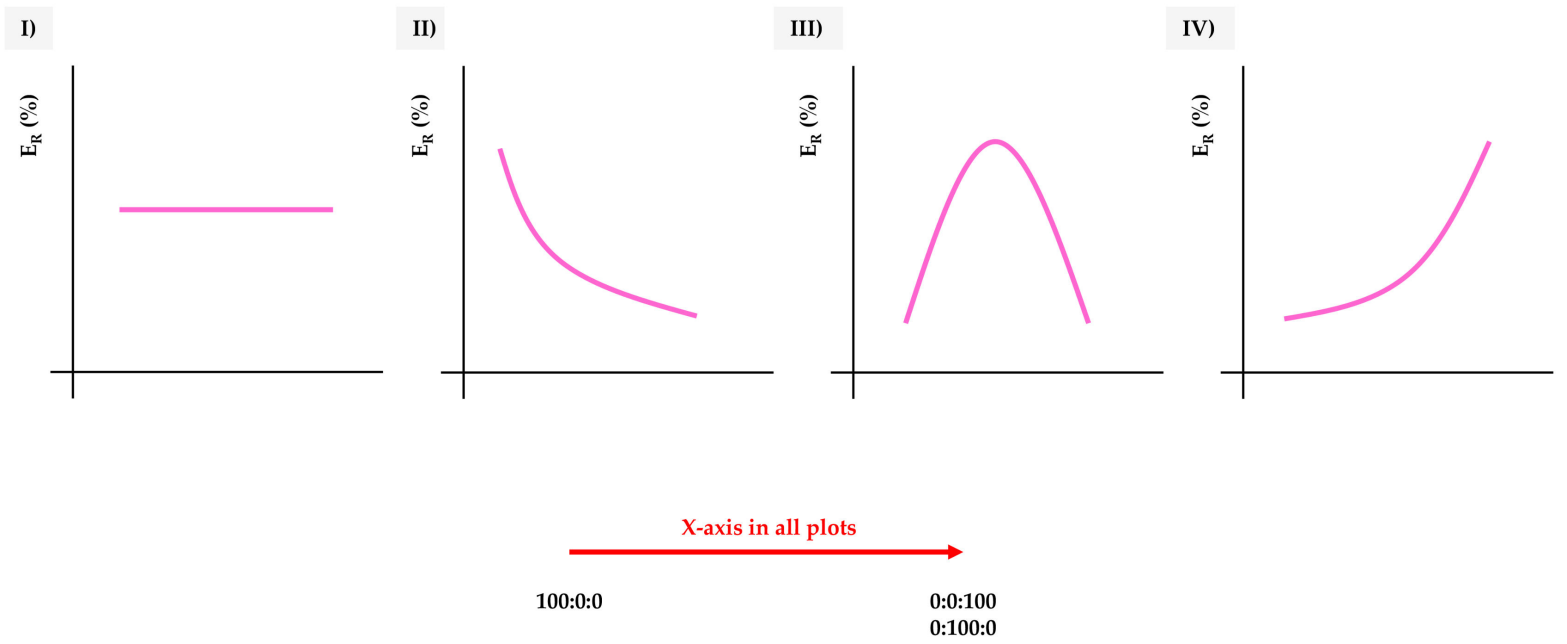
Types of general trends observed when extracting the target analytes by dispersive miniaturized solid-phase extraction (D-µSPE)-high-performance liquid chromatography (HPLC)-diode array detection (DAD) using different UiO-66-based MOFs as sorbents, as a function of the extraction efficiency (E_R_ in %). X-axis goes generically from the neat UiO-66 (H-bdc), 100:0:0, to increasing amounts of functionalization, to reach 0:0:100 (NO_2_-bdc) or 0:100:0 (NH_2_-bdc).

If taking the extraction efficiency (of the microextraction method) as the main feature to evaluate the influence of the MOFs’ nature in the method, four main trends can be observed in this study, as summarized in Figure 3. The extraction efficiency (E_R_) was calculated as the ratio of the real enrichment factor of the microextraction procedure (calculated in the analytical method) and the theoretical maximum enrichment factor (40) [12], and expressed as %.

In trend I, there is no variation in the extraction efficiency, independently of the degree and type of functionalization. This means that the analyte is not interacting with the functional groups introduced, and it is not affected by the decrease of the pore window of the MOFs.

Trend II shows a decrease in the extraction efficiency with an increase in the functionalization. This situation is attributable to analytes with a critical size, highly affected by the decrease in the MOF’s pore window, or to analytes able to establish strong interactions with the functional groups, thus precluding a good desorption process from the MOF once trapped (Figure S1 of the ESM).

Trend III shows a maximum at intermediate degrees of functionalization, with extraction efficiencies achieved with the neat MOFs lower than those achieved for the mixed ones. In this case, the analytes benefit from both types of functionalization, for example, larger window aperture due to bare terephthalic and hydrogen bond interactions caused by amino-modified ligands.

Trend IV is indeed the opposite to trend II: the functionalization increases the extraction efficiency of the analyte by the MOF. In this case, the window aperture is not a problem for the analyte and the interactions established with the functional groups increase the adsorption capability of the material.

#### 3.2.1. Analytical Performance When Using the H-UiO-66 to NO_2_-UiO-66 Series

Figure S7 of the ESM shows a comparison of the extraction efficiency for all the analytes when the amount of nitro functionalization in terephthalate ligands of the MOF increases. In general, the extraction efficiencies are better when using an intermediate degree of functionalization, indicating that the mixed-ligand approach produces small, but significant, improvements with respect to the bare UiO-66 MOF. Trend III is particularly clear in the case of carbamazepine, triclosan, and benzophenone-3, which are small-size analytes able to penetrate into the pores, and their polar groups (amide in Cbz, hydroxyl in Tr and BP-3) are able to establish hydrogen bond interactions with the nitro group of the MOF.

Chy, Ind, and OP also present better extraction efficiencies when using mixed-ligand UiO-66 MOFs, although the variation is less pronounced than for the smaller analytes. The size of these analytes is at the limit of the window aperture of the UiO-66 pores, and most likely, their adsorption occurs on the external surface of the crystallites. The addition of functional groups to the terephthalic ligands reduces the window aperture, and therefore, the small increase in the extraction efficiency may come from interactions with the nitro groups on the external surface of the UiO-66 crystallites.

#### 3.2.2. Analytical Performance When Using the H-UiO-66 to NH_2_-UiO-66 Series

The extraction efficiency for all the analytes when increasing the number of amino groups in the UiO-66 MOF is depicted in Figure S8 of the ESM. In this case, there are a variety of trends. For Cbz, there is an increase (trend IV) in the extraction efficiency with the increase in amino groups, indicating that the capability of the MOF to establish hydrogen bonds may be critical for this analyte. The amide–amino interaction is most likely responsible for this better E_R._ A similar trend (IV) is observed for BP-3, where the acceptor groups of the benzophenone-3 can establish H-bonds with the donor amino groups of the MOF.

Furthermore, CuP and Tr show a continuous decrease in E_R_ values when the amount of amino groups increases (trend II). It seems that the presence of functional groups does not favor the extraction recovery. If we consider that the two analytes have similar structural features (two benzene rings and hydroxyl groups), this trend is most likely due to the limitation in the pore window size and the presence of polar groups in the pore walls.

As occurs for the nitro series, Chy and Ind experience better recoveries with mixed MOFs, with 50:50:0 functionalization being the one that produces the best results. In the case of the OP and t-OP analytes, they show different trends; for t-OP, the E_R_ drops after 50% of amino-terephthalic incorporation, and for OP, the E_R_ rises somewhat when the number of amino groups increases.

#### 3.2.3. Analytical Performance When Using the NH_2_-bdc to NO_2_-bdc Series

The trends for the extraction efficiencies when a mixture of nitro- and amino-terephthalic ligands are used in the synthesis of UiO-66 are shown in Figure S9 of the ESM. In this case, it seems that the mixture clearly favors the recovery of the analytes, since in all the cases, except for OP, the E_R_ is better when using mixed-ligand MOFs. The reasons may not be the same for all the analytes, but the introduction of some amino groups (less bulky, H-bond donor) produces a better environment for analyte adsorption. In some cases, although a significant enhancement of the E_R_ is produced (BP-3, Chy, and Ind) with 0:25:75, the increase of amino groups content does not imply a better E_R_.

### 3.3. Study of the Analytical Performance of the D-µSPE-HPLC-DAD Method Focusing on the Analyte’s Structure and Possible MOF–Analyte Interactions

The individual analysis carried out by series (Section 3.2) gives a limited vision of the influence of functionalization in UiO-66. If intending the extraction of a specific target analyte by D-µSPE-HPLC-DAD, it is important to have an overview to obtain semi-quantitative conclusions related to which functionalization in the MOF is best for an individual analyte in the method.

**Figure 4:**
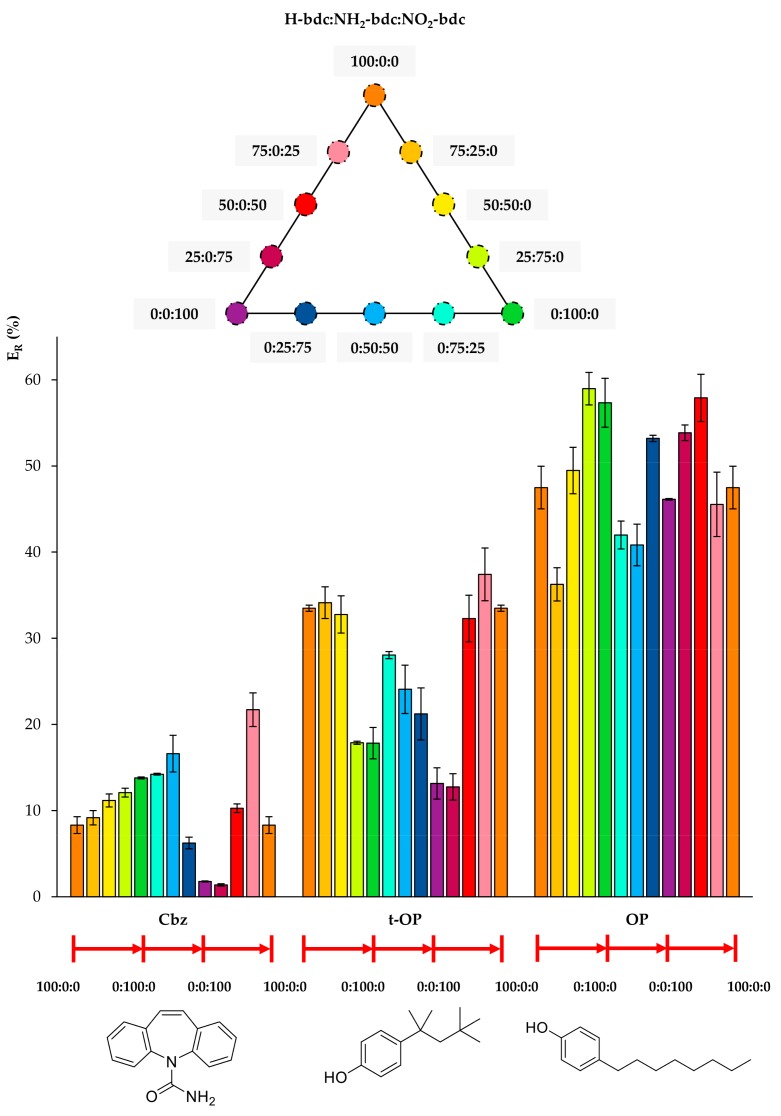
Extraction efficiencies (E_R_ in %) using the twelve UiO-66-based MOFs as sorbents in D-µSPE-HPLC-DAD for the analytes carbamazepine (Cbz), 4-tert-octylphenol (t-OP), and 4-octylphenol (OP) (selected for presenting similar structures).

**Figure 5:**
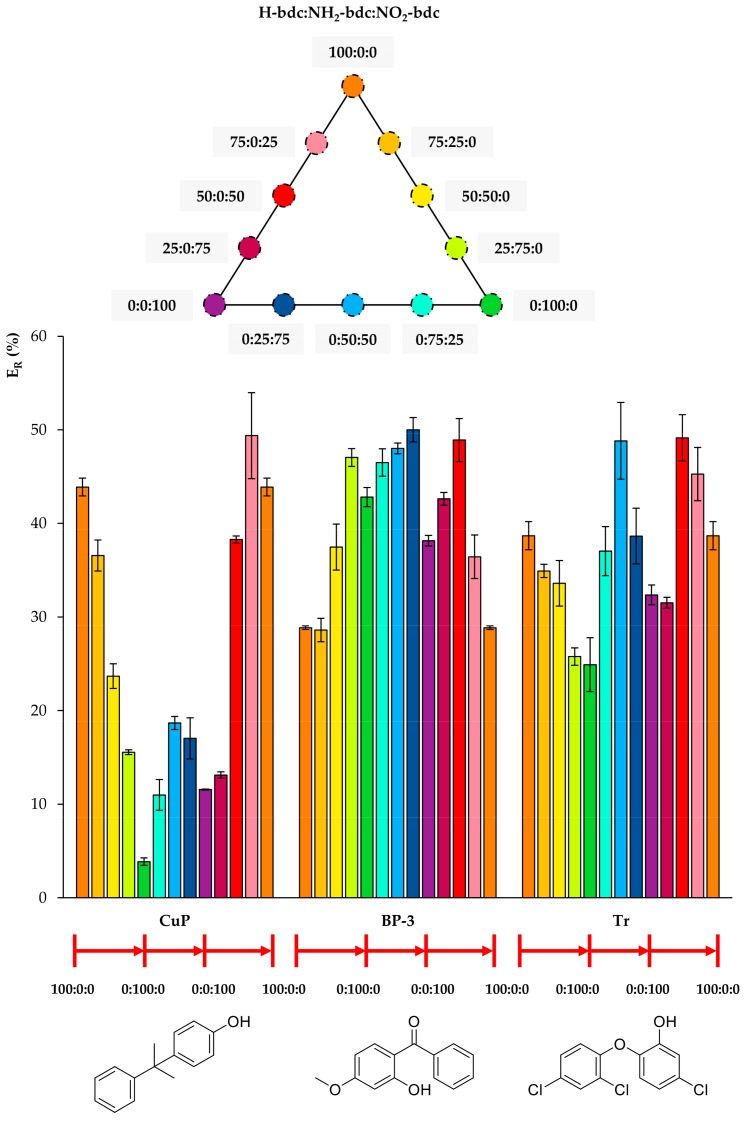
Efficiencies (E_R_ in %) using the twelve UiO-66-based MOFs as sorbents in D-µSPE-HPLC-DAD for the analytes 4-cumylphenol (CuP), benzophenone-3 (BP-3), and triclosan (Tr) (selected for presenting similar structures).

**Figure 6:**
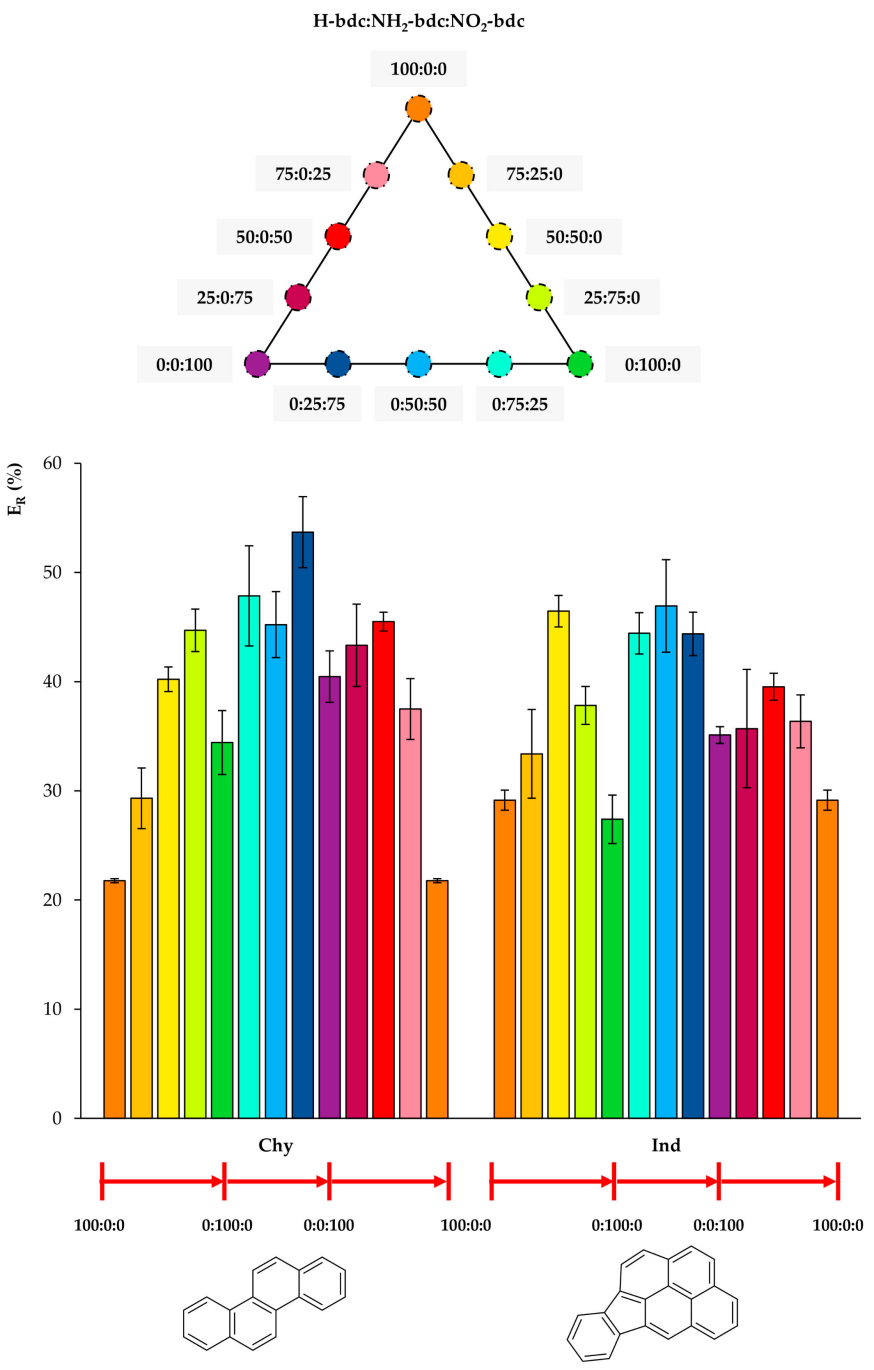
Extraction efficiencies (E_R_ in %) using the twelve UiO-66-based MOFs as sorbents in D-µSPE-HPLC-DAD for the analytes chrysene (Chy) and indeno(1,2,3-cd)pyrene (Ind) (selected for presenting similar structures).

Figure 4, Figure 5 and Figure 6 include the extraction efficiency achieved with each of the twelve MOFs tested as sorbents using D-µSPE-HPLC-DAD, grouped in three different plots as a function of structural similarities of analytes. This gives a general overview by the analyte’s nature when using all MOFs. It is important to note that all studies were accomplished using the eight analytes present all together in the aqueous standard subjected to the entire method, and thus, effects in the E_R_ values coming from analyte–analyte interactions could have occurred.

Regarding Cbz, Figure 4 shows that the best performance occurs when there is a slight inclusion of NO_2_ functionalization in the bare H-bdc UiO-66. Although the nitro group seems to favor the extraction of Cbz, the further increase in nitro groups produces a decrease in E_R_, indicating that another effect, most likely the decrease of the pore window, reduces the diffusion through the pores. In the case of the t-OP, the inclusion of functional groups in the UiO-66 decreases the extraction performance, with the best values being obtained for MOFs with little or no functionalization. For OP, its extraction efficiencies fluctuate, but without a clear trend, and the best performances are obtained using 0:100:0 (neat NH_2_-UiO-66) and 50:0:50 (mixed nitro and bare UiO-66).

Figure 5 includes the behavior of CuP, which is similar to that of t-OP, but even more drastic. Its E_R_ value using the MOF 100% NH_2_-bdc UiO-66 in D-µSPE-HPLC-DAD is lower than 4%, but when using the neat UiO-66 without any functional group, it is higher than 40%. Although CuP exhibits a hydroxyl H-bond donor, the functionalization of the MOF with polar groups is not key for its extraction, and it is possible that the decrease of pore aperture plays a more significant role. In the case of BP-3, the incorporation of both NH_2_ and NO_2_ functional groups in the UiO-66 MOF produces better E_R_ values. The best performance is achieved using 0:25:75 NH_2_/NO_2_ mixed UiO-66. The presence of H-bond acceptor and donor groups in the same MOF favors the extraction of BP-3, an analyte with H-bond donor (hydroxyl) and acceptor (ketone) groups. For Tr, the best performance occurs when utilizing 0:50:50 and 50:0:50 UiO-MOFs, indicating that the presence of nitro groups contributes to more efficient extractions, but the excess of this group produces a final decrease in E_R_. As it has been abovementioned, the combination of amino and nitro groups has good effects on the overall extraction performance of the method for the analyte.

In Figure 6, it can be observed that any functionalization of the bare UiO-66 increases the extraction efficiency for Chy, and the best performances occur for mixed NH_2_/NO_2_ UiO-66. Regarding Ind, similarly to Chy, the functionalization of UiO-66 produces better E_R_ values, and the 50:50:0, 0:50:50 mixed UiO-66 led to the best values.

## 4. Conclusions

The mixed-ligand strategy has been demonstrated to be a useful and simple tool for incorporating different functionalization groups and thus different pore environments into the MOF UiO-66. The strategy permitted the tailoring of UiO-66, and the resulting MOFs containing a variety of polar functional groups showed a high efficiency for the microextraction of target pollutants from waters when used as sorbents in D-µSPE.

Different trends were observed in the obtained extraction efficiency, depending on the structure of the target analyte and on the type and degree of functionalization of UiO-66. Therefore, the presence of H-bond donor groups in UiO-66 improves the analytical performance of the D-µSPE-HPLC-DAD method for those target compounds containing groups in their structures able to participate in H-bonds. However, the presence of polar groups in UiO-66 can significantly reduce the extraction efficiency for analytes with bulky hydrophobic groups in their structures. Clearly, proper control of the structure of the MOF is possible by carefully considering the type of target analytes intended.

It is not possible to quantitatively estimate the amount of amino and nitro groups to be included in UiO-66 to ensure an improved efficiency for the entire group of target analytes selected in the current study. However, the use of mixed amounts of both groups in UiO-66 seems to represent an adequate selection.

Ongoing studies intend to investigate the application of this mixed functionalization strategy to other MOFs, in order to develop tailored analytical microextraction methods.

## Figures and Tables

**Table 1:** Several physicochemical properties of the analytes studied (SciFinder^®^ 2019).

Analyte (Abbreviation)	Structure	Molecular Formula Molecular Weight (g·mol^−1^)	Molar Volume ^1^ (Å^3^·molecule^−1^)	pK_a_	Vapor Pressure at 25 ^°^C (N·m^−2^)	Log K_ow_^2^
Carbamazepine (Cbz)	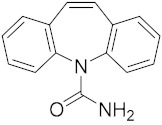	C_15_H_12_N_2_O 236.27	310	13.9	7.71 × 10^−5^	1.90
4-Cumylphenol (CuP)	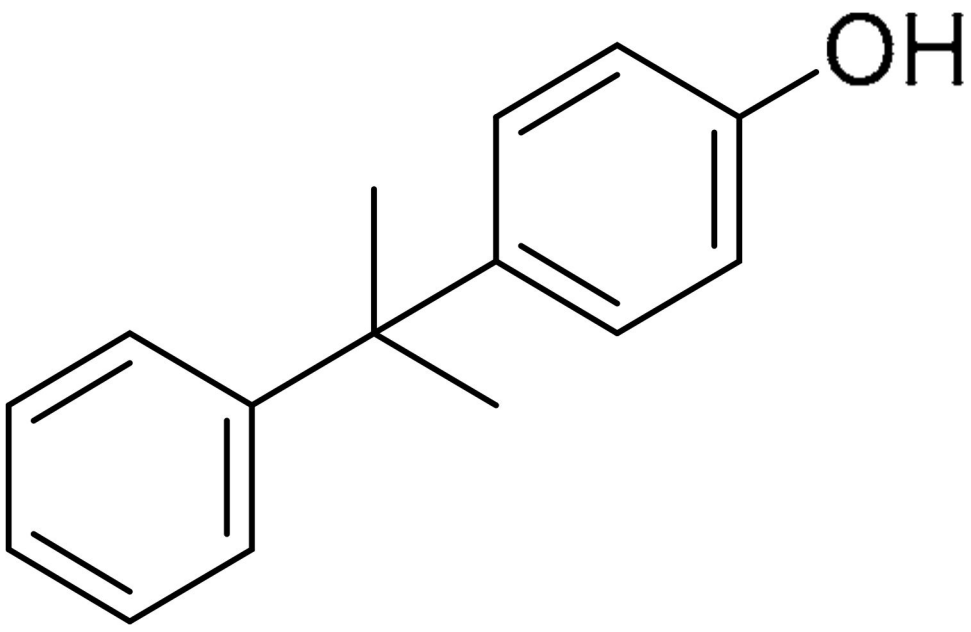	C_15_H_16_O 212.29	334	10.6	6.64 × 10^−3^	4.24
Benzophenone-3 (BP-3)	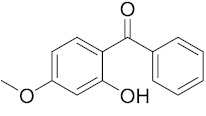	C_14_H_12_O_3_ 228.24	315	7.6	7.01 × 10^−4^	4.00
Triclosan (Tr)	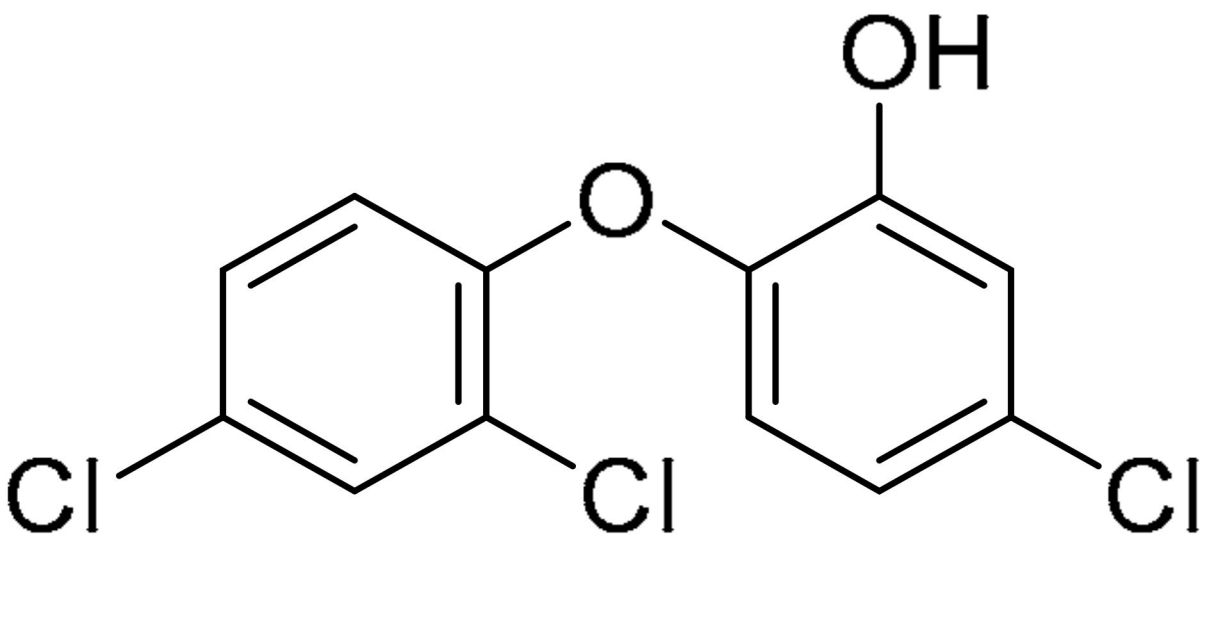	C_12_H_7_Cl_3_O_2_ 289.54	323	7.8	4.35 × 10^−3^	5.34
4-*tert*-Octylphenol (t-OP)	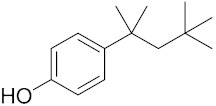	C_14_H_22_O 206.32	366	10.2	2.64 × 10^−1^	5.18
4-Octylphenol (OP)	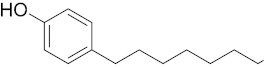	C_14_H_22_O 206.32	365	10.2	3.33 × 10^−2^	5.63
Chrysene (Chy)	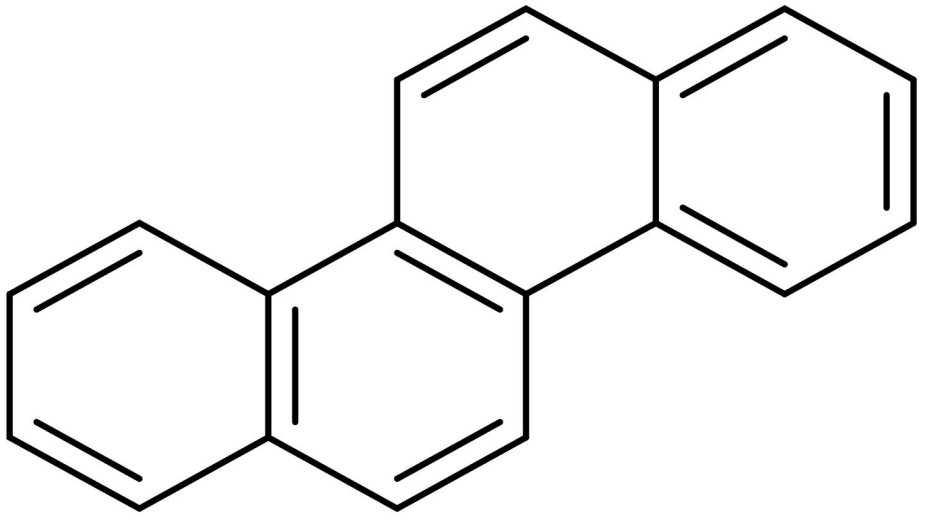	C_18_H_12_ 228.29	318	-	1.13 × 10^−5^	5.73
Indeno[1,2,3-cd]pyrene (Ind)	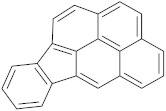	C_22_H_12_ 276.33	333	-	2.08 × 10^−7^	6.65

^1^ 20 °C and 1.01·10^5^ N·m^−2^;^2^ octanol/water partition coefficient.

## Supplementary Materials

The following are available online, Figure S1: Scheme of the D-μSPE-HPLC-DAD method using optimum conditions; Figure S2–S4: XRD patterns; Figure S5, S6: Infrared spectra; Figure S7–S9: Analytical performances; Table S1: Adsorption data for all the synthesized UiO-66-based MOFs; Table S2: Elemental analysis data for all the synthesized UiO-66-based MOFs.
