# Supplementary material for: Mixed Functionalization of Organic Ligands in UiO-66: A Tool to Design Metal–Organic Frameworks for Tailored Microextraction
Source: Molecules. 2019 Oct 10;24(20):3656. doi: 10.3390/molecules24203656 (PMC6832283; doi:10.3390/molecules24203656)
Supplement: Supplementary file 1 [file molecules-24-03656-s001.pdf]

Supplementary Material

Mixed functionalization of organic ligands in UiO-66: a tool to design metal-organic frameworks for tailored microextraction

Gabriel González-Rodríguez[1], Iván Taima-Mancera[1], Ana B. Lago[2], Juan H. Ayala[1], Jorge Pasán[2,*], Verónica Pino[1,3,]**

[1]*Departamento de Química, Unidad Departamental de Química Analítica, Universidad de La Laguna (ULL), La Laguna, Tenerife, 38206 Spain*

[2]*Laboratorio de Rayos X y Materiales Moleculares (MATMOL), Departamento de Física, Universidad de La Laguna (ULL), La Laguna, Tenerife, 38206 Spain*

[3]*University Institute of Tropical Diseases and Public Health, Universidad de La Laguna (ULL), Tenerife, 38206 Spain*

Table of contents

FIGURES

Figure S1 .... page S1

Figure S2 .... page S2

Figure S3 .... page S3

Figure S4 .... page S4

Figure S5 .... page S5

Figure S6 .... page S6

Figure S7 .... page S7

Figure S8 .... page S8

Figure S9 .... page S9

TABLES

Table S1 .... page S10

Table S2 .... page S11

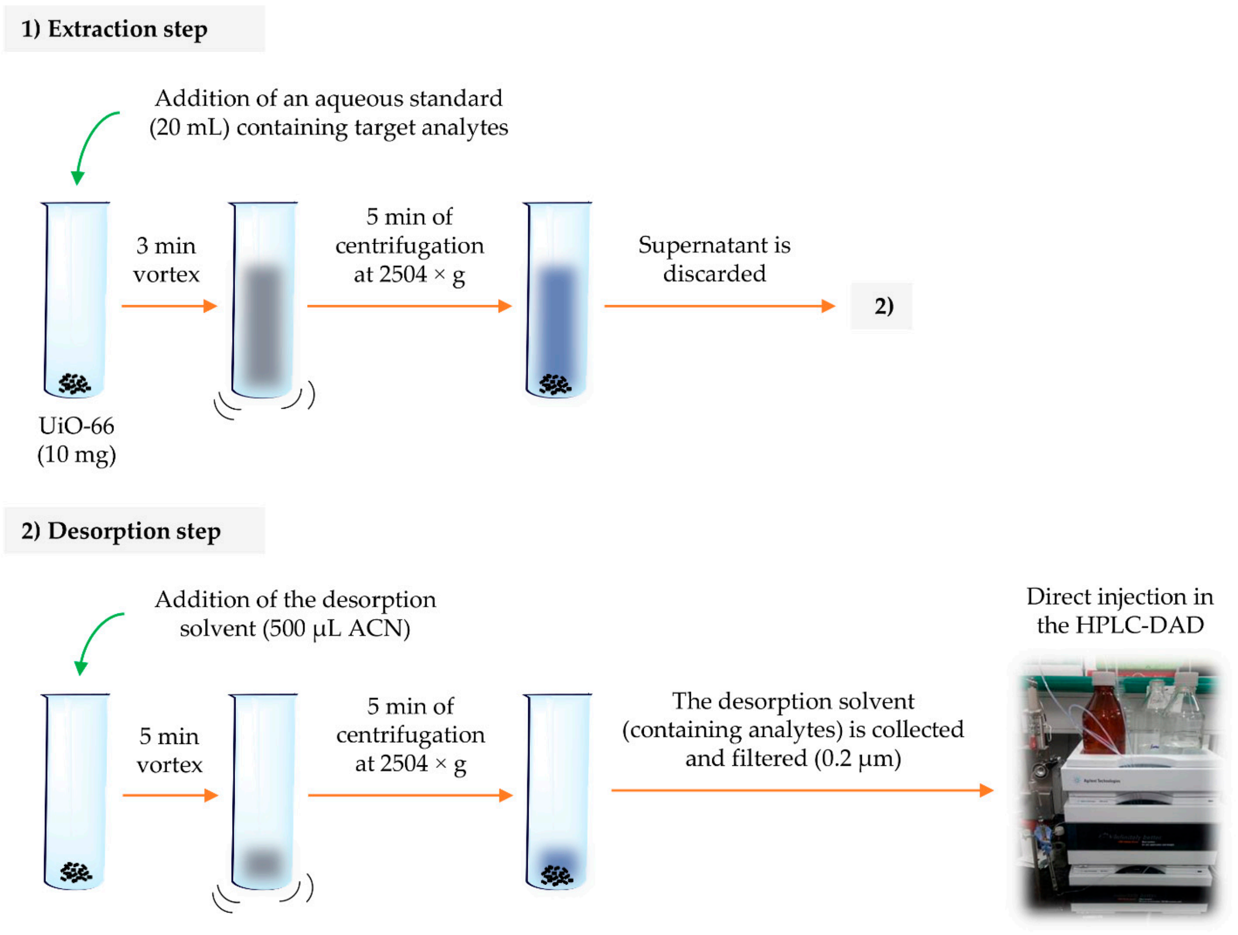

Figure S1. Scheme of the D-µSPE-HPLC-DAD method using optimum conditions.

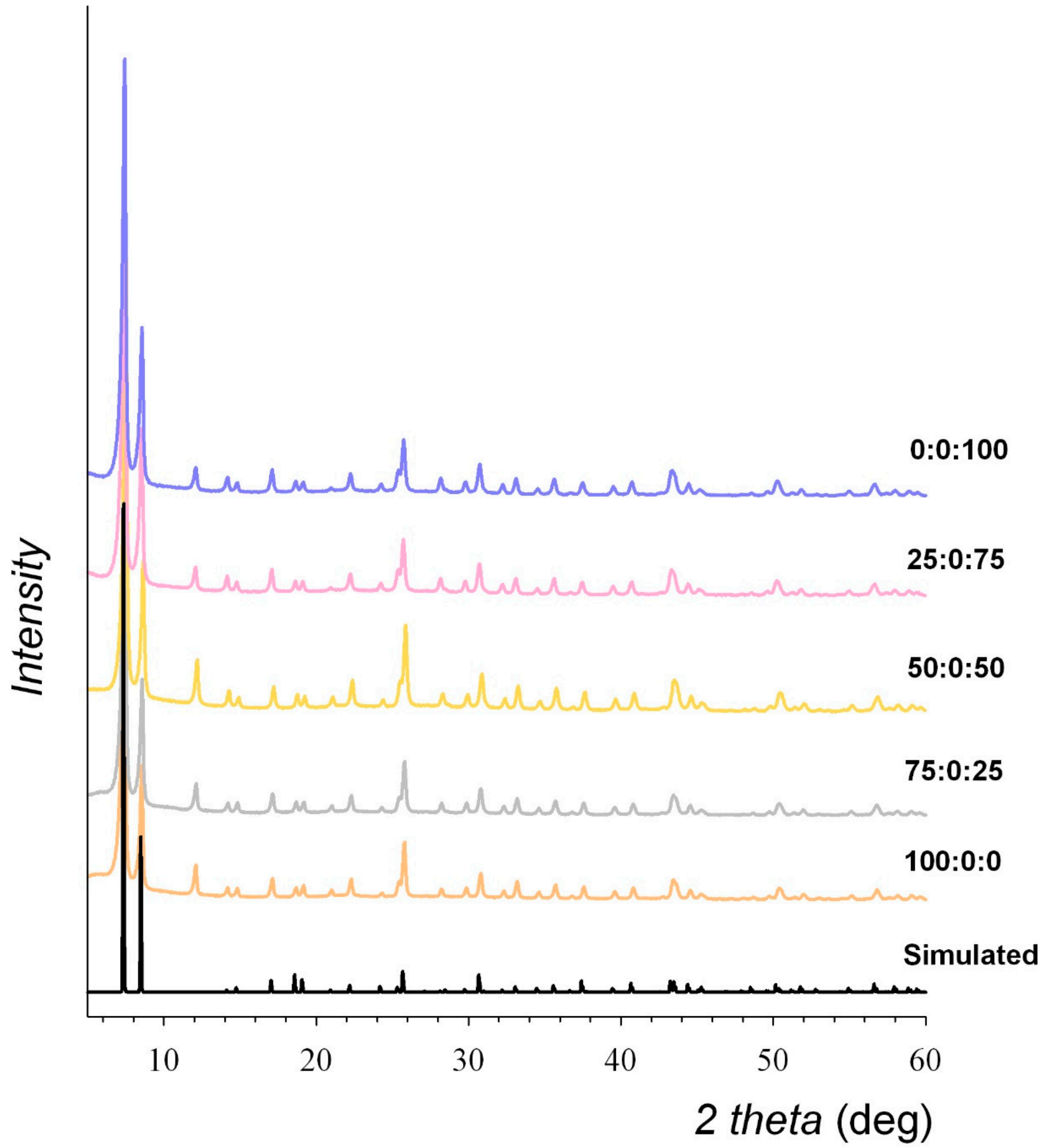

Figure S2. XRD patterns of the H-bdc/NO_2-bdc series of UiO-66.

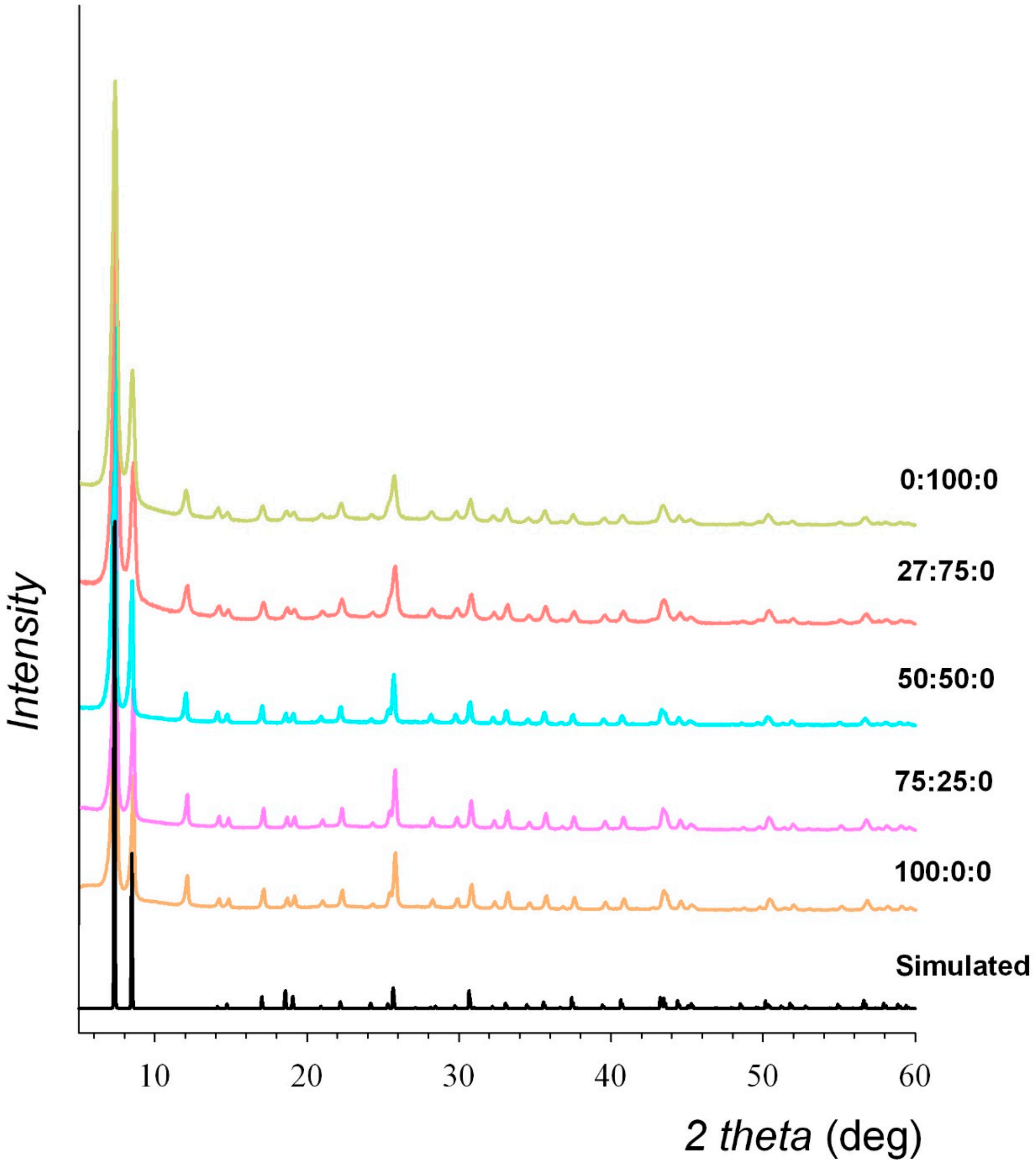

Figure S3. XRD patterns of the H-bdc/NH_2-bdc series of UiO-66.

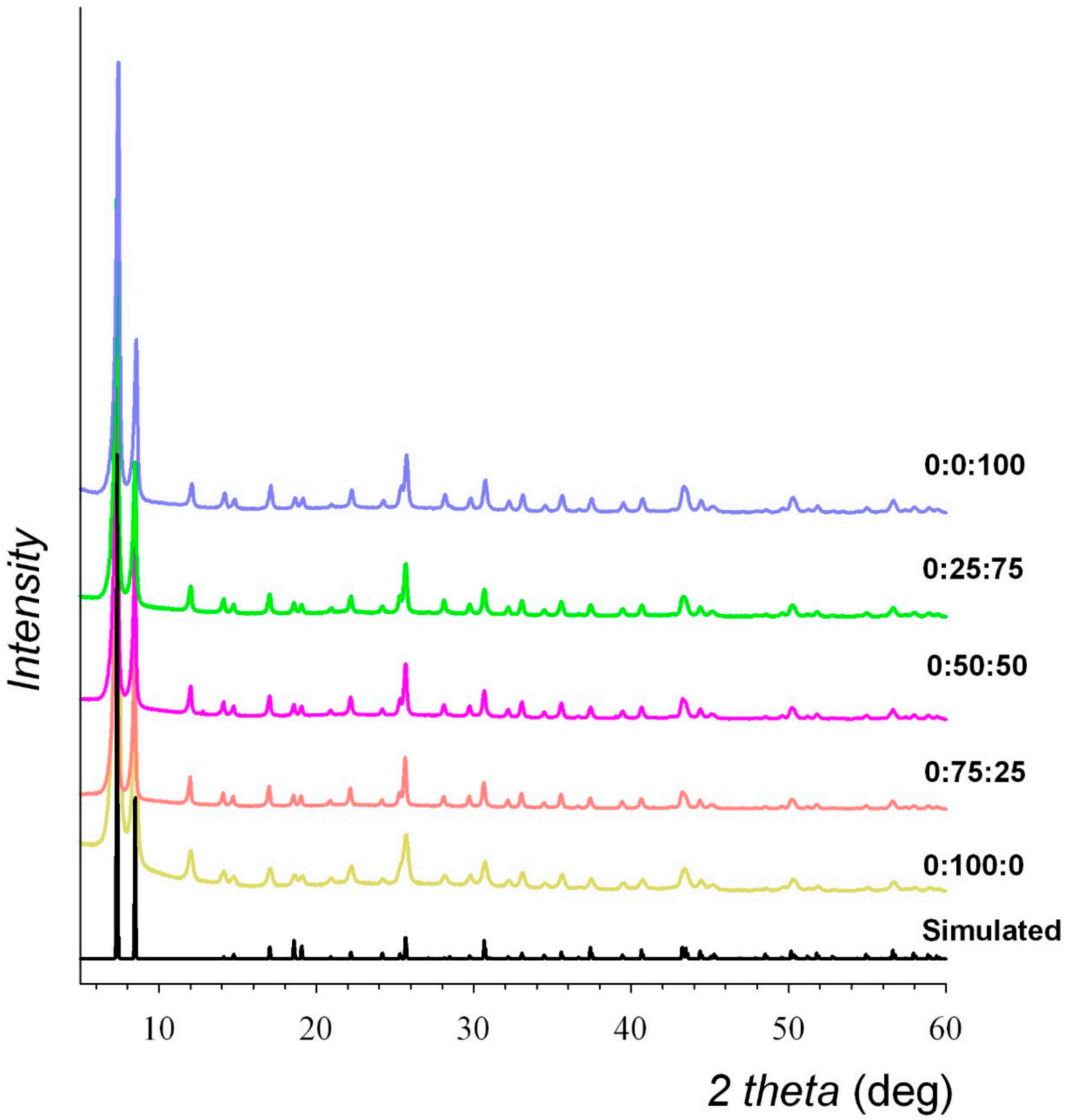

Figure S4. XRD patterns of the NH_2-bdc/NO_2-bdc series of UiO-66.

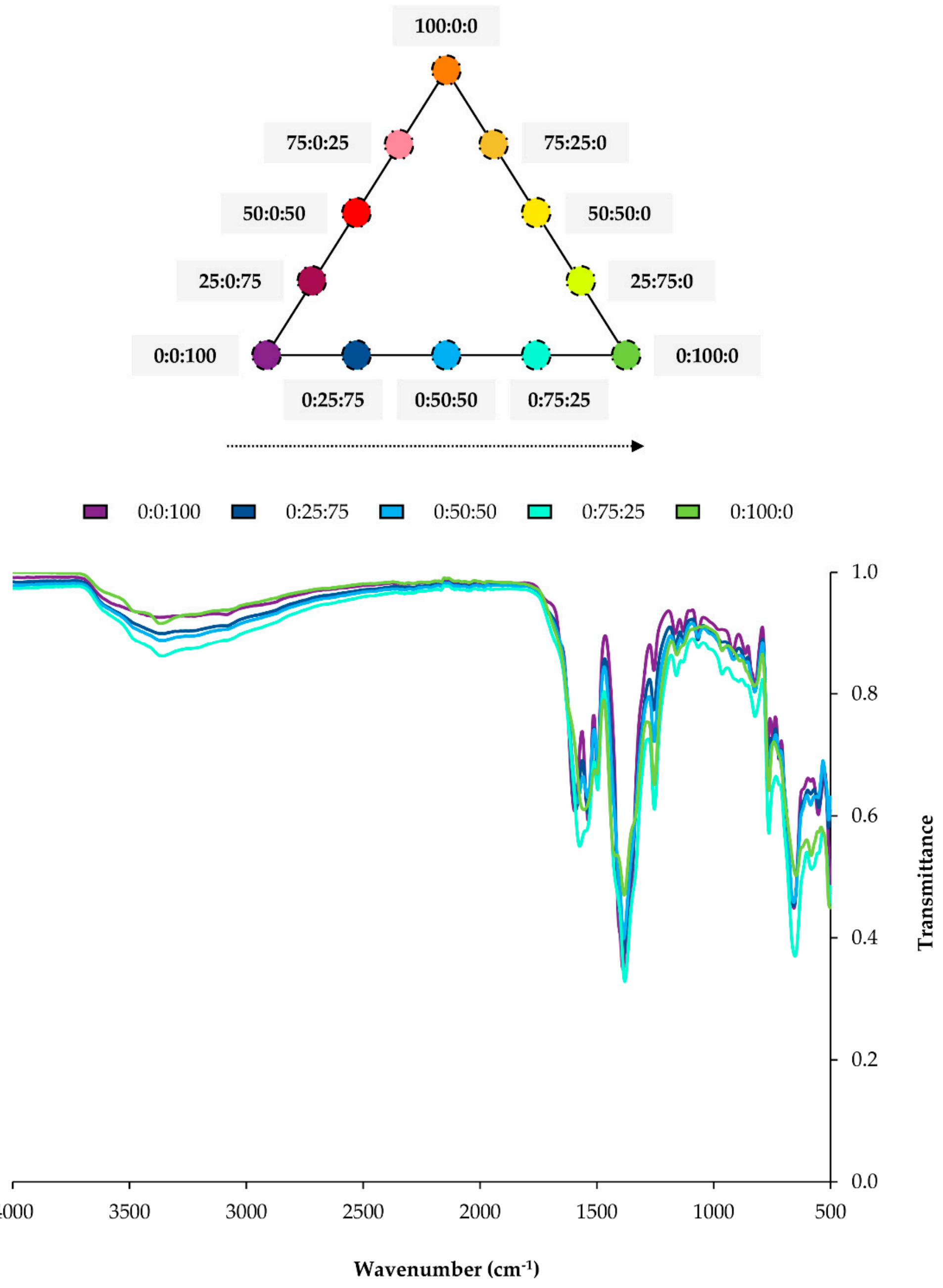

Figure S5. Infrared spectra for the NH_2-bdc/NO_2-bdc series. The code H-bdc:NH_2-bdc:NO_2-bdc is included for each MOF.

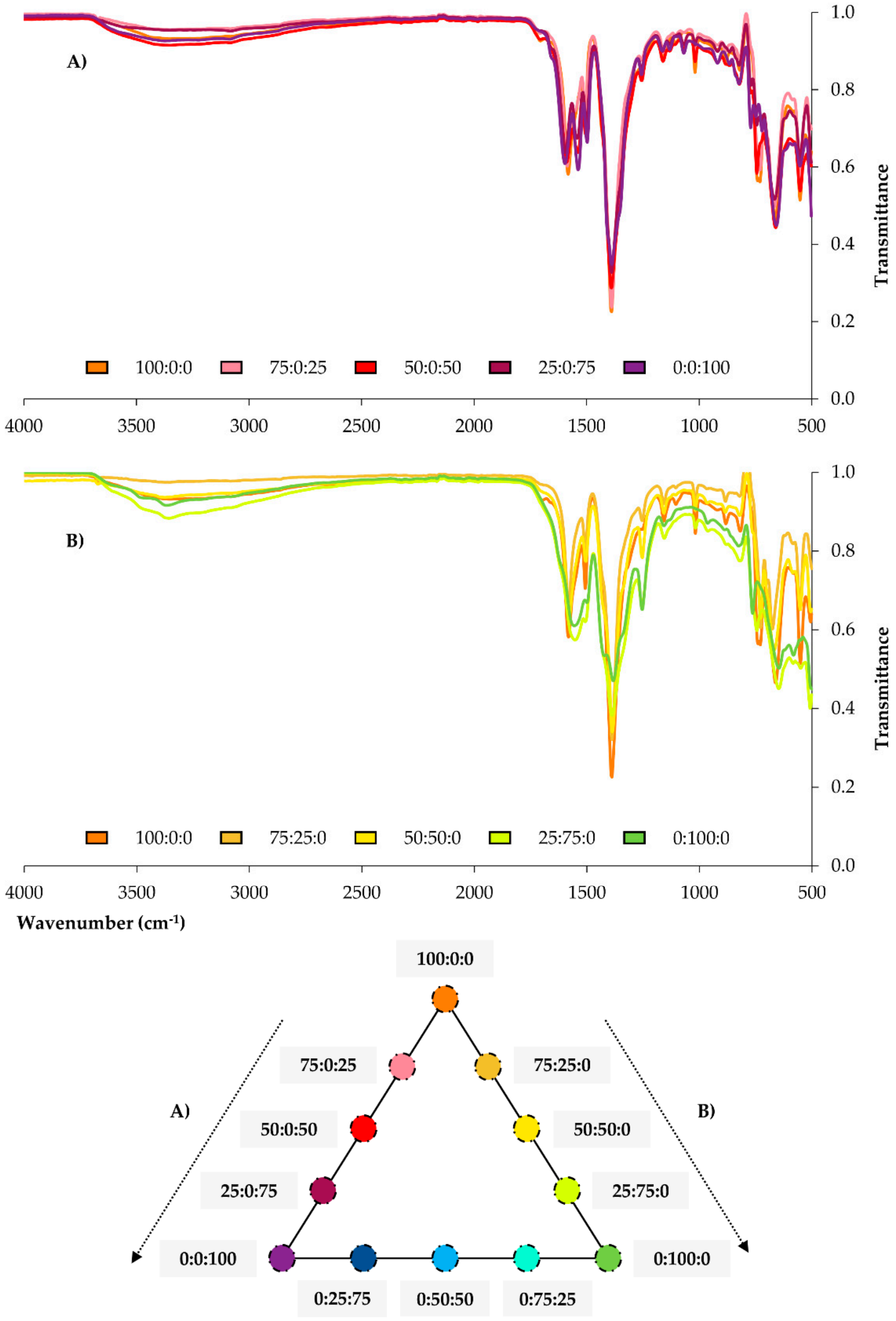

Figure S6. Infrared spectra for **A)** H-bdc/NO_2-bdc and **B)** H-bdc/NH_2-bdc series. The code H-bdc:NH_2-bdc:NO_2-bdc is included for each MOF. The small band at around 1700 cm^{-1} in the FTIR can be linked to DMF moieties retained inside the porous structure strongly adsorbed.

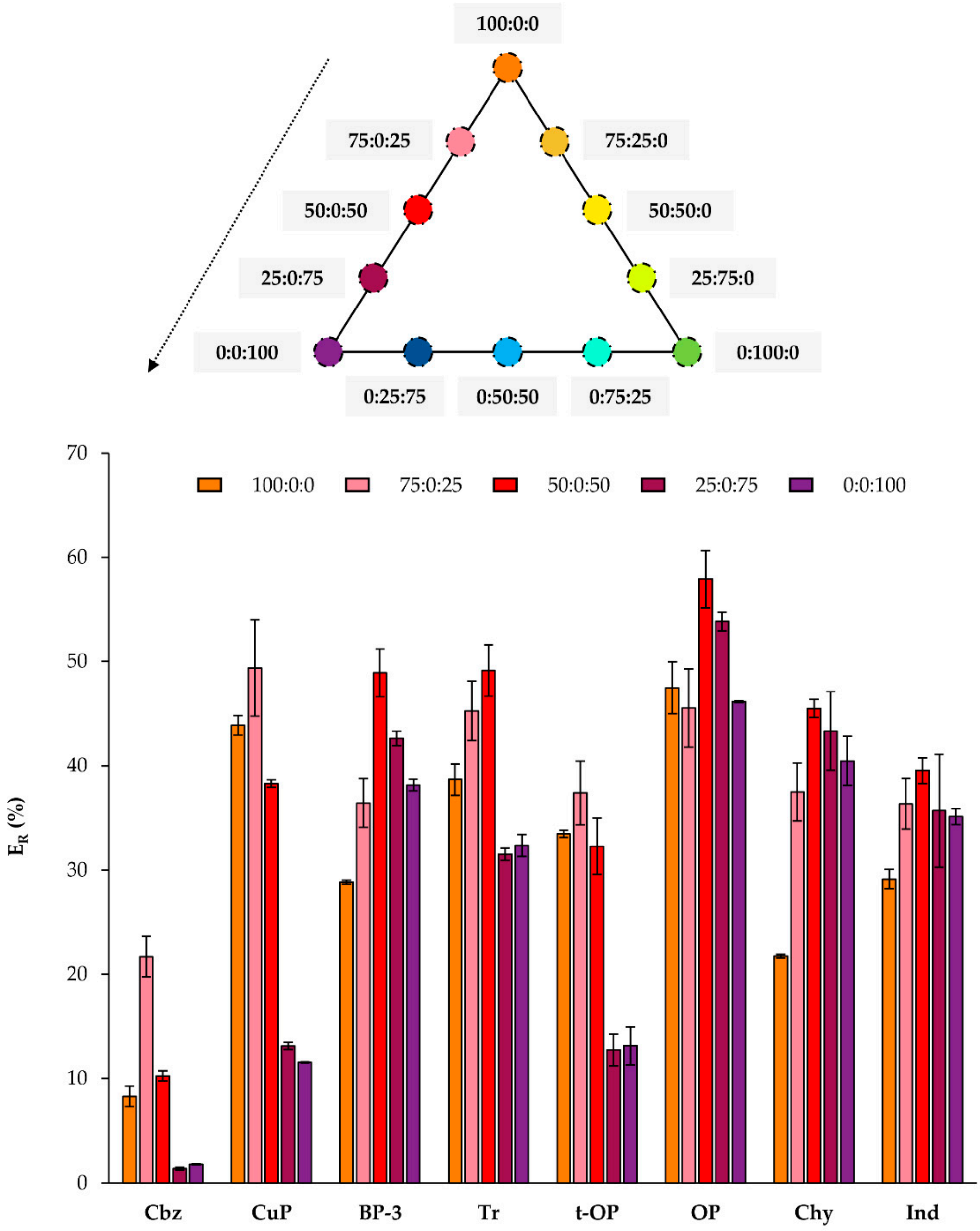

Figure S7. Analytical performance when using the H-bdc/NO_2-bdc series of UiO-66 as sorbents in D-µSPE-HPLC-DAD, expressed in terms of extraction efficiency (E_R, %) for the target analytes. The code H-bdc:NH_2-bdc:NO_2-bdc is included for each MOF. Experiments were performed in triplicate, with the conditions described in section 2.3.2.

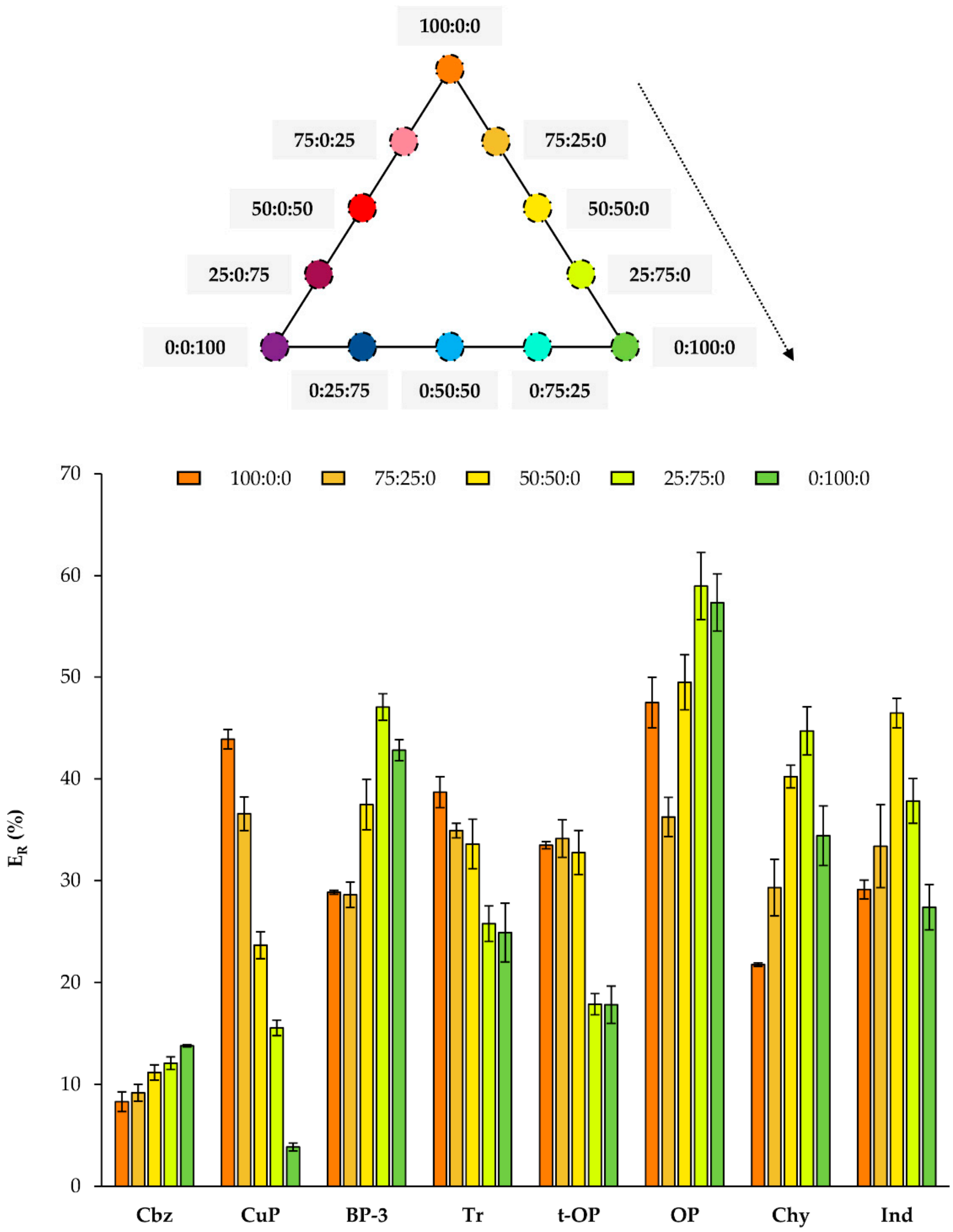

Figure S8. Analytical performance when using the H-bdc/NH_2-bdc series of MOFs as sorbents in D-µSPE-HPLC-DAD, expressed in terms of extraction efficiency (E_R, %) for the target analytes. The code H-bdc:NH_2-bdc:NO_2-bdc is included for each MOF. Experiments were performed in triplicate, with the conditions described in section 2.3.2.

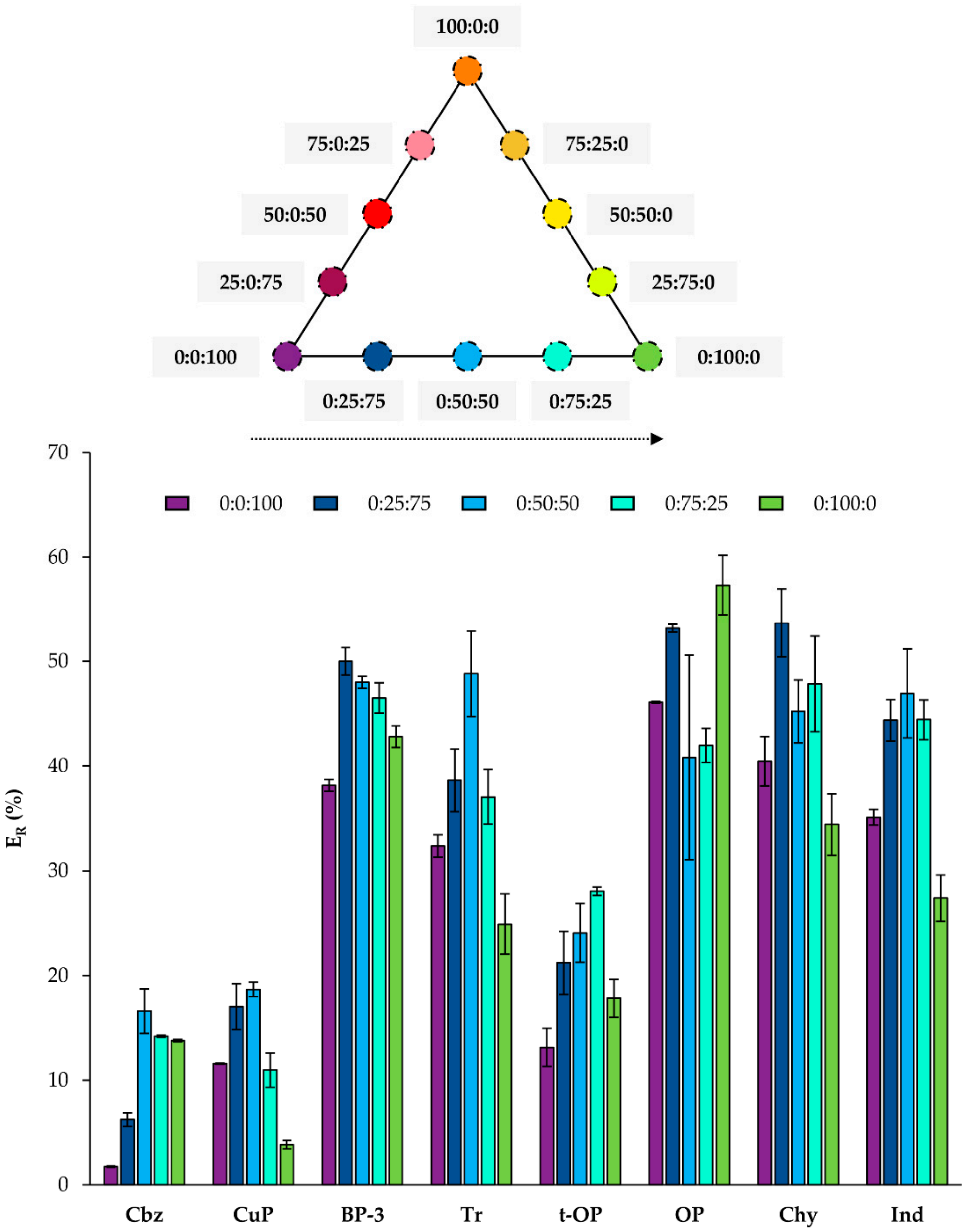

Figure S9. Analytical performance when using the NH_2-bdc/NO_2-bdc series of MOFs as sorbents in D-µSPE-HPLC-DAD, expressed in terms of extraction efficiency (E_R, %) for the target analytes. The code H-bdc:NH_2-bdc:NO_2-bdc is included for each MOF. Experiments were carried out in triplicate, with the conditions described in section 2.3.2.

Table S1. Adsorption data for all the synthesized UiO-66-based MOFs.

MOF (H-bdc:NH_2-bdc:NO_2-bdc)	Surface area	
	BET[1] ($m^2 \cdot g^{-1}$)	Langmuir[2] ($m^2 \cdot g^{-1}$)
100:0:0	1175	1480
75:25:0	938	1312
50:50:0	895	1444
25:75:0	757	1050
0:100:0	678	939
75:0:25	717	991
50:0:50	689	953
25:0:75	656	845
0:0:100	604	752
0:25:75	620	802
0:50:50	684	889
0:75:25	719	924

[1] Brunauer-Emmett-Teller multilayer gas adsorption model

[2] Langmuir monolayer gas adsorption model

Table S2. Elemental analysis data for all the synthesized UiO-66-based MOFs.

MOF (H-bdc:NH_2-bdc:NO_2-bdc)	Experimental / Calculated (%)		
	C	N	H
100:0:0	32.09 / 32.86	0.24 / 0.00	2.09 / 2.18
75:25:0	31.59 / 32.45	1.58 / 1.18	2.03 / 2.24
50:50:0	31.31 / 31.72	3.09 / 2.31	2.93 / 2.28
25:75:0	29.95 / 31.65	3.67 / 3.46	2.89 / 2.35
0:100:0	30.05 / 30.96	4.48 / 4.51	2.45 / 2.49
75:0:25	30.73 / 31.65	1.57 / 1.15	1.90 / 2.01
50:0:50	29.41 / 30.51	2.61 / 2.22	1.69 / 1.87
25:0:75	29.69 / 29.46	3.83 / 3.22	1.71 / 1.72
0:0:100	28.13 / 28.48	4.47 / 4.15	1.85 / 1.59
0:25:75	28.99 / 29.12	4.58 / 4.25	1.54 / 1.78
0:50:50	29.16 / 29.80	4.46 / 4.35	1.98 / 1.98
0:75:25	29.88 / 30.51	4.45 / 4.44	2.20 / 2.19
